# Supplementary material for: Benchmarking Community-Wide Estimates of Growth Potential from Metagenomes Using Codon Usage Statistics
Source: mSystems. 2022 Oct 3;7(5):e00745-22. doi: 10.1128/msystems.00745-22 (PMC9600850; doi:10.1128/msystems.00745-22)
Supplement: TABLE S1 [file msystems.00745-22-s0001.pdf]

Model	CUB Metrics	Other Variables	CUB Calculated at the Single-Gene Level
MMBC*	Δ MILC	GC, OGT	Y
MMv1*	$MILC_{HE}$	OGT	N
growthpred	ΔENC', S	OGT	Y
gRodon “full”	$MILC_{HE}$	Ψ, Codon Pair Bias, OGT	N
